# Validation of the new classification criteria for systemic lupus erythematosus on a patient cohort from a national referral center: a retrospective study

**DOI:** 10.3325/CroatMedJ_60_0325

**Published:** 2019-08

**Authors:** Marija Bakula, Nada Čikeš, Branimir Anić

**Affiliations:** 1 Division of Clinical Immunology and Rheumatology, Department of Internal Medicine, University Hospital Center Zagreb, University of Zagreb School of Medicine, Zagreb, Croatia; 2 Center for Translational and Clinical Research, University of Zagreb School of Medicine and University Hospital Center Zagreb, Zagreb, Croatia

## Abstract

**Aim:**

To validate Systemic Lupus International Collaborating Clinics (SLICC)-12 and American College of Rheumatology (ACR)-97 classification criteria on a patient cohort from the University Hospital Center Zagreb.

**Methods:**

This retrospective study, conducted from 2014 to 2016, involved 308 patients with systemic lupus erythematosus (SLE) (n = 146) and SLE-allied conditions (n = 162). Patients' medical charts were evaluated by an expert rheumatologist to confirm the clinical diagnosis, regardless of the number of the ACR-97 criteria met. Overall sensitivity and specificity, as well as the sensitivity and specificity according to disease duration, were compared between ACR-97 and SLICC-12 classifications. Predictive value for SLE for both classifications was assessed using logistic regression and receiver operating characteristic (ROC) curves.

**Results:**

The SLICC-12 criteria had significantly higher sensitivity in early disease, which increased with disease duration. The ACR-97 criteria had higher specificity. The specificity of the SLICC-12 criteria was low and decreased with disease duration. Regression analysis demonstrated the superiority of the SLICC-12 classification criteria over the ACR-97 criteria, with areas under the ROC curve of 0.801 and 0.780, respectively.

**Conclusion:**

Although the SLICC-12 criteria were superior to the ACR-97 and were more sensitive for diagnosing early SLE, their specificity in our population was too low. The sensitivity of the SLICC-12 classification is increased by better defined clinical features within each criterion. Our results contribute to the current initiative for developing new criteria for SLE.

Systemic lupus erythematosus (SLE) is a chronic autoimmune disease characterized by the production of autoantibodies and immune complexes, and their deposition into different tissues. Its etiology is still unclear, with genetic and environmental factors contributing to disease development (1-3). SLE has a relapsing-remitting course, and disease damage accumulates over time. Given that the period from the disease onset to permanent damage can be long, SLE is hard to diagnose in its early stages. It is even more difficult to classify patients using classification criteria of the American College of Rheumatology (ACR). Therefore, in a clinical setting, substitute entities such as incomplete lupus erythematosus (ILE), preclinical lupus, or latent lupus are often mentioned when describing patients whose clinical and laboratory findings suggest SLE but who meet fewer than 4 ACR criteria (4-6).

Advances in understanding of pathogenetic mechanisms require the redefinition and validation of classification criteria. Redefined criteria would encompass some of the patients with undefined disease and enable more thorough monitoring of the disease course and more prompt therapy introduction. In rheumatology, the development of classification criteria is challenging due to the lack of a clinical, etiopathogenetic, or diagnostic gold standard. Instead, the gold standard remains the expert rheumatologist's opinion (7,8).

ACR published the first, preliminary, criteria for SLE in 1971, which were revised in 1982 and later validated. The second revision from 1997 (ACR-97), although unvalidated, has been the most widely used criteria set to date (9-13) (Table 1). The prerequisite for enrolling patients in clinical studies is cumulative attainment of any 4 of the 11 ACR criteria. Despite their high sensitivity (>85%) and specificity (>95%) in longstanding SLE, their sensitivity in early disease can be significantly lower (14-16). Furthermore, some organ systems are overrepresented (eg, 4 criteria attributed to skin and mucosa), with equal contribution of every criterion to the diagnosis regardless of its specificity and sensitivity. Clinical experience and previous studies have shown greater statistical significance of certain criteria, such as renal impairment, discoid rash, or cytopenias (14,17,18). Different statistical methods have been employed with the purpose to develop optimal classification rule (19-22). Somogy et al validated the ACR criteria on a patient cohort treated at the University Hospital Centre (UHC) Zagreb in 1993 (18), demonstrating substantial differences in the weight of individual criteria. Cerovec et al reported the prevalence of ACR-97 criteria in patients treated at UHC Zagreb (23).

**Table 1:** Difference between Systemic Lupus International Collaborating Clinics (SLICC)-12 and American College of Rheumatology (ACR)-97 classification criteria*

SLICC 2012	ACR 1997
ACL (maculopapulous, psoriasiform, bullous, toxic epidermal necrolysis)	Malar rash
CCL (hypertrophic, mucosal, panniculitis, chilblain, LE tumidus, discoid/lichen planus overlap)	Discoid rash
Nonscarring alopecia	Photosensitivity
Oral ulcers (also *ex anamnesis*)	Oral ulcers (present at check-up)
Joint-line tenderness, morning stiffness, also erosive	Arthritis (non-erosive)
Pleuritis, pericarditis	Serositis (pleuritis, pericarditis)
Renal disorder (daily OR spot urine – protein/creatinine ratio, 24h proteinuria >0.5 g, cellular casts, red blood cell casts)	Renal disorder (daily protein/creatinine ratio, 24h proteinuria >0.5g, cellular casts)
Seizures, psychosis, M.multiplex, myelitis, periph/cran neuropathy, acute confusional state	Psychosis, grand mal epilepsy
Hemolytic anemia Leukopenia (<4000 mm^3^)/Lymphopenia (<1000 mm^3^) – ONCE Thrombocytopenia <100,000/mm^3^ – ONCE	Hemolytic anemia OR on ≥2 occasions: Leukopenia <4000/mm^3^ OR Lymphopenia <1000/mm^3^ OR Thrombocytopenia <100000/mm^3^
Anti-dsDNA Anti-Sm ACL Ig-A/M/G, LAC, RPR, anti-β2 GPI (Ig-A/M/G) Low C3, C4, CH 50 Direct Coombs+ (absence of hemolytic anemia) ANA	Anti-dsDNA OR Anti-Sm OR ACL IgG/IgM, LAC, RPR
	ANA

*ACL –acute cutaneous lupus; CCL – chronic cutaneous lupus; LE – lupus erythematosus; dsDNA – double stranded DNA; anti-Sm – anti-Smith antibody; aCL – anticardiolipins; LAC – lupus anticoagulant; RPR – rapid plasma reagin test; anti-β2 GPI – anti-β2-glycoprotein I; C3 – complement component 3; C4 – complement component 4; CH50 – 50% hemolytic complement activity; ANA – anti-nuclear antibody.

Systemic Lupus International Collaborating Clinics (SLICC) group in 2012 published a new classification based on revised ACR-97 criteria, comprising 17 criteria (11 clinical and 6 immunologic, Table 1) (14). The SLICC-12 classification, more thoroughly defines each criterion, encompasses some of the typical mucocutaneous manifestations and neuropsychiatric symptoms omitted in the ACR-97 classification, alters arthritis definition, and significantly revises laboratory findings. SLICC-12 classification specifies that patients can be diagnosed with SLE if they meet 4 of the 17 criteria with at least 1 immunologic and 1 clinical criterion. One of the most important improvements is that biopsy-proven lupus nephritis with positive antinuclear antibody (ANA) and/or anti-double stranded DNA (dsDNA) is a sufficient requirement to meet the SLE diagnosis.

The multi-annual revision of the SLE classification conducted by the SLICC was a two-step process consisting of criteria derivation and criteria validation in two large patient groups. In the derivation group, the SLICC-12 classification showed greater sensitivity than the ACR-97 criteria, almost equal specificity, and had significantly fewer missclassifications. In the validation step there was no statistical difference between the two classifications (14).

Studies published to date have shown that the SLICC-12 classification successfully recognizes patients with established disease. Furthermore, the ACR-97 and SLICC-12 criteria are complementary in discriminating SLE from ILE with a milder disease course (24,25). The first studies have reported higher sensitivity of the SLICC-12 classification compared with the ACR-97 criteria in the early stages of SLE (6,26). The SLICC-12 criteria need to be further evaluated and validated on new cohorts to investigate if they are superior to ACR-97 classification.

Division of Clinical Immunology and Rheumatology, UHC Zagreb, Croatia, is a national referral center for SLE and lupus-allied diseases. The Division serves patients from all over the country, predominantly from northwestern Croatia. The aim of this research was (i) to validate the new SLICC-12 classification criteria in our patient cohort, in a real-life setting, (ii) to compare the ACR-97 and SLICC-12 criteria and asses if SLICC-12 criteria recognize patients in early stages of SLE (<5 years) better than the ACR-97 criteria, and (iii) to form a well-defined patient cohort with SLE for future research using the SLICC-12 criteria with the purpose of distinguishing patients with early-onset SLE.

## Patients and methods

### Patients

This retrospective study comprised 308 patients treated at the Division's outpatient clinic. From 2010 to 2012, a complete patient database at the Division of Clinical immunology and Rheumatology was reviewed and patient documentation assessed. The database contained data on approximately 30 000 patients, 2000 of whom had SLE, SLE-allied, and other unspecified conditions (collagenosis, antiphospholipid syndrome [APS], Sy Sjögren, mixed connective tissue disease [MCTD], undifferentiated connective tissue disease [UCTD], discoid lupus erythematosus [DLE], Sy Raynaud, glomerulonephritis, overlap syndrome, oligoarthritis HLAA1B8DR3 positive, lymphopenia). Out of these 2000 patients, 527 patients were defined, followed up, and clinically diagnosed with SLE or suspected SLE. They were the starting point for conducting this research. The study also included 201 new patients with SLE or potential SLE who started their follow up at the Department from 2012 to 2016. The patients with insufficient medical data or without regular follow ups were excluded. Finally, the patients were divided in two groups: 146 patients with established SLE (diagnosed by rheumatologist) and 162 patients with SLE-allied conditions. The criterion for the study inclusion was not the number of classification criteria fulfilled, but the clinical diagnosis determined by the expert rheumatologist, which remains the gold standard for diagnosing SLE.

Patients who had to undergo kidney biopsy for lupus nephritis staging have signed an informed consent, which is a standard procedure at the Clinic. The study was approved by the Ethics Committee of the University Hospital Center Zagreb.

### Methods

All of the patient records were reevaluated by expert rheumatologist in order to determine if they agree with the diagnosis. For every patient, a check-list of SLE-related features was filled out. The association between clinical diagnosis and diagnosis generated on the basis of both ACR-97 and SLICC-12 classification criteria was assessed. The overall sensitivity and specificity of ACR-97 and SLICC-12 classifications, as well as the sensitivity and specificity according to disease duration was calculated. The predictive value of every criterion in ACR-97 and SLICC-12 classification was assessed using logistic regression analysis and receiver operating characteristic (ROC) curves.

Laboratory tests were carried out at the UHC Zagreb. ANA were determined by indirect immunofluorescence on Hep-2 cells (Euroimmun, Lübeck, Germany). Specific antibodies against dsDNA, histones, Sjögren-Syndrom-related antigen A and B, Smith, U1-ribonucleoprotein, scleroderma-70, and histidyl-tRNA synthetase were detected by fluorescent color-coded beads immunotest – Luminex (AtheNA-ANA multiplex assay, Zeus Scientific Inc., Branchburg, NJ, USA). Autoantibodies against different phospholipid antigen determinants (IgM and IgG anticardiolipin antibodies and IgG anti- β2 glycoprotein I antibodies) were determined by enzyme-linked immunosorbent assay (Orgentec Diagnostics GmbH, Mainz and Euroimmun, Lübeck, Germany). Finally, hemolytic activity of the classical activation pathway was quantified by determining serum dilution causing lysis of 50% sheep erythrocytes covered with sheep IgM antibodies (CH50, *in house* method).

### Statistical analysis

Simple descriptive statistics was computed in Microsoft Excel 2010 (Microsoft Corporation, Redmond, WA, USA), while univariate and multivariate logistic regression and stepwise procedure were performed in SAS (SAS Institute, Cary, NC, USA) with the purpose to determine the best predictive models (27). A 5% level of significance (type I error) was considered significant. Differences in proportions were assessed using the test of proportions.

## Results

### Disease duration and sex distribution

Mean disease duration from the time of first symptoms' onset was 11 ± 7 years, while the mean disease duration from the time of diagnosis was 10 ± 7 years. The relatively short period needed to diagnose SLE could be attributed to prompt and effective diagnostic procedure in a highly specialized tertiary institution. Our cohort consisted of 279/308 (90.6%) female patients, which is the expected sex distribution in SLE patients (1-3). Sex distribution was almost equal in two patient-groups: 132 (90.4%) women in the SLE-group and 147 (90.7%) women in the NSLE-group. Mean age of all patients was 51 ± 14 years (range 20-88). Male patients had somewhat lower age (mean 48 ± 13) than female patients (mean 52 ± 15). The mean age at the first visit was the same as the age at the time of diagnosis (41 ± 14 years).

### Prevalence of SLICC-12 and ACR-97 classification criteria

The prevalence of both SLICC-12 and ACR-97 classification criteria was assesed in both SLE and NSLE patient group (Tables 2 and 3). Since NSLE patients have diseases similar to SLE, with overlapping clinical symptoms, we identified the criteria that were significantly more represented in the SLE-group. As expected, all of the criteria from the SLICC-12 and ACR-97 classification were more prevalent in the SLE-group. Among SLICC-12 criteria, significantly more prevalent were renal impairment (11.6% in SLE-group vs 3.7% in NSLE-group, *P* = 0.008), serositis (6.9% in SLE-group vs 1.2% in NSLE-group, *P* = 0.010), and synovitis (39.7% in SLE-group vs 28.5% in NSLE-group, *P* = 0.038). Furthermore, each of the 6 SLICC-12 immunologic criteria was significantly more prevalent in the SLE-group (Table 3). Among ACR-97 criteria, significantly more prevalent were renal impairment (12.3% in SLE-group vs 3.7% in NSLE-group, *P* = 0.005), serositis (5.5% in SLE-group vs 1.2% in NSLE-group, *P* = 0.033), immunologic criterion (80.1% in SLE-group vs 42.3% in NSLE-group, *P* < 0.0001), and ANA antibodies (94.5% in SLE-group vs 84.6% in NSLE-group, *P* = 0.005).

**Table 2:** Prevalence of the ACR-97 criteria in SLE (N = 146) and NSLE (N = 162) patient groups*

ACR criteria	Total, n (%)	SLE, n (%)	NSLE, n (%)	*P* (SLE vs NSLE)
**Photosensitivity**	53 (17.2)	27 (18.5)	26 (16)	0.562
**Butterfly rash**	49 (15.9)	26 (17.8)	23 (14.2)	0.389
**Discoid lupus**	37 (12)	18 (12.3)	19 (11.7)	0.871
**Oral ulcerations**	8 (2.6)	4 (2.7)	4 (2.5)	0.912
**Arthritis**	80 (26)	39 (26.7)	41 (25.3)	0.780
**Serositis**	10 (3.2)	8 (5.5)	2 (1.2)	0.033
**Renal impairment**	24 (7.8)	18 (12.3)	6 (3.7)	0.005
**NPSLE**	9 (2.9)	6 (4.1)	3 (1.8)	0.229
**Hematologic criterion**	144 (46.7)	69 (47.3)	75 (46.3)	0.860
**Immunologic criterion**	186 (60.4)	117 (80.1)	69 (42.3)	<0.0001
**ANA**	275 (89.3)	138 (94.5)	137 (84.6)	0.005

*SLICC-12 – Systemic Lupus International Collaboration Clinics classification from 2012; ACR-97 – American College of Rheumatology classification from 1997; NSLE – nonSLE; patients with SLE-allied conditions; NPSLE – neuropsychiatric lupus erythematosus; ANA – anti-nuclear antibody.

**Table 3:** Prevalence of the SLICC-12 criteria in SLE (N = 146) and NSLE (N = 162) patient groups*

SLICC criteria	Total, n (%)	SLE, n (%)	NSLE, n (%)	*P* (SLE vs NSLE)
**ACL**	100 (32.5)	48 (33)	52 (32)	0.851
**CCL**	34 (11)	17 (11.6)	17 (10.5)	0.758
**Alopecia**	23 (7.5)	12 (8.2)	11 (6.8)	0.641
**Oral ulcerations**	12 (3.9)	7 (4.8)	5 (3)	0.411
**Synovitis**	104 (33.7)	58 (39.7)	46 (28.5)	0.038
**Serositis**	12 (3.9)	10 (6.9)	2 (1.2)	0.010
**Renal impairment**	23 (7.5)	17 (11.6)	6 (3.7)	0.008
**NPSLE**	40 (12.9)	21 (14.4)	19 (11.7)	0.482
**Hemolytic anemia**	7 (2.3)	7 (4.58)	0	N/A
**Leuko/lymphopenia**	130 (42.2)	63 (43.2)	67 (41.4)	0.749
**Thrombocytopenia**	37 (12)	18 (12.3)	19 (11.7)	0.871
**ANA**	275 (89.3)	136 (92.5)	139 (85.8)	0.030
**Anti-dsDNA**	155 (50)	103 (70.5)	52 (32.1)	<0.0001
**Anti-Sm**	37 (12)	23 (15.7)	14 (8.6)	0.055
**APA**	103 (33.4)	65 (44.5)	38 (23.5)	0.0001
**Complement**	140 (45.4)	76 (52)	64 (39.5)	0.028
**Coombs test**	10 (3.3)	8 (5.5)	2 (1.2)	0.033

*SLICC-12 – Systemic Lupus International Collaboration Clinics classification from 2012; ACR-97 – American College of Rheumatology classification from 1997; NSLE – nonSLE; patients with SLE-allied conditions; ACL –acute cutaneous lupus; CCL – chronic cutaneous lupus; NPSLE – neuropsychiatric lupus erythematosus; ANA – anti-nuclear antibody; dsDNA – double stranded DNA; anti-Sm – anti-Smith antibody; APA – anti-phospholipid antibody; N/A – not applicable.

### Sensitivity and specificity of SLICC-12 and ACR-97 criteria by disease duration

**Figure 1:**
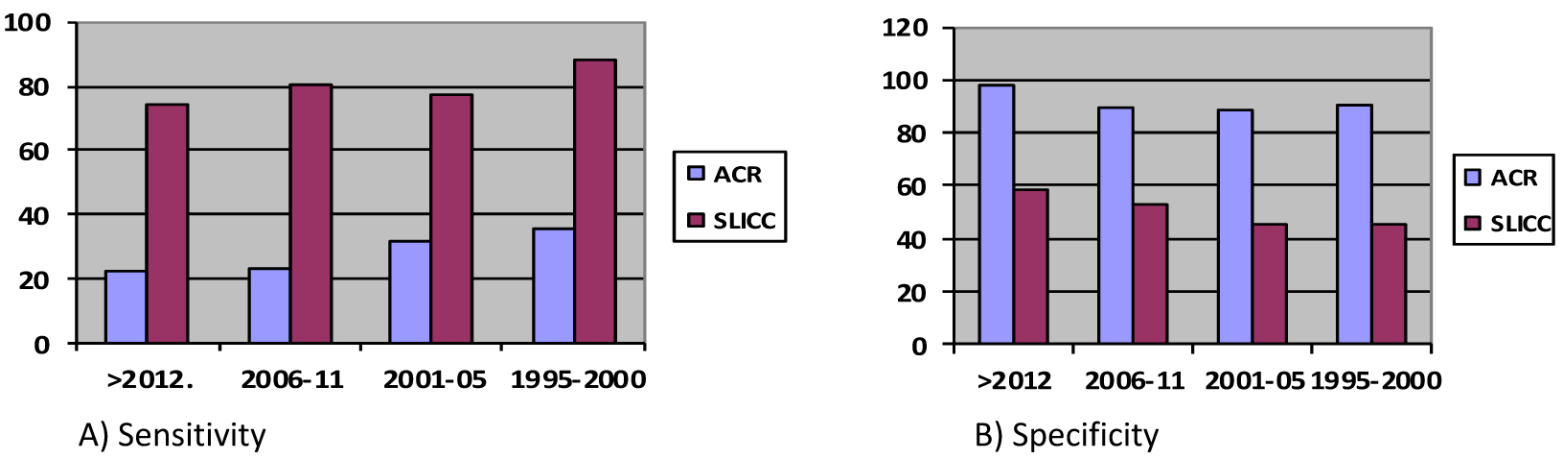
Sensitivity (**A**) and specificity (**B**) of Systemic Lupus International Collaborating Clinics (SLICC)-12 (red) and American College of Rheumatology (ACR)-97 classification criteria (blue) by disease duration.

In order to assess if SLICC-12 classification was superior to ACR-97 classification in earlier stages of SLE, we distributed the patients into groups according to disease duration: the early disease group (disease onset max. 5 years ago) and the remaining groups by five-year periods of disease duration (5-10 years, 10-15 years, 15-20 years, 20-25 years, and 25-30 years). In the early-disease group, the SLICC-12 criteria had 74% sensitivity and a low specificity of 58.5%. The sensitivity and specificity of the ACR-97 criteria were 22.2% and 98.1%, respectively. The sensitivity of the SLICC-12 criteria increased with disease duration, while their specificity decreased. On the other hand, the low sensitivity of the ACR-97 criteria in the early SLE stage showed a slow, but stabile growth. Similarly, their specificity was high in all patient groups and increased to 100% in the long-standing, established disease (Figure 1A and 1B)

### Regression analysis of SLICC-12 and ACR-97 classification criteria

Using regression analysis, we tested the predictive value of classification criteria for diagnosing SLE in our patient cohort. Additionally, to select the best combination of classification criteria and to create the most adequate model for predicting SLE, we employed stepwise procedure.

**Figure 2:**
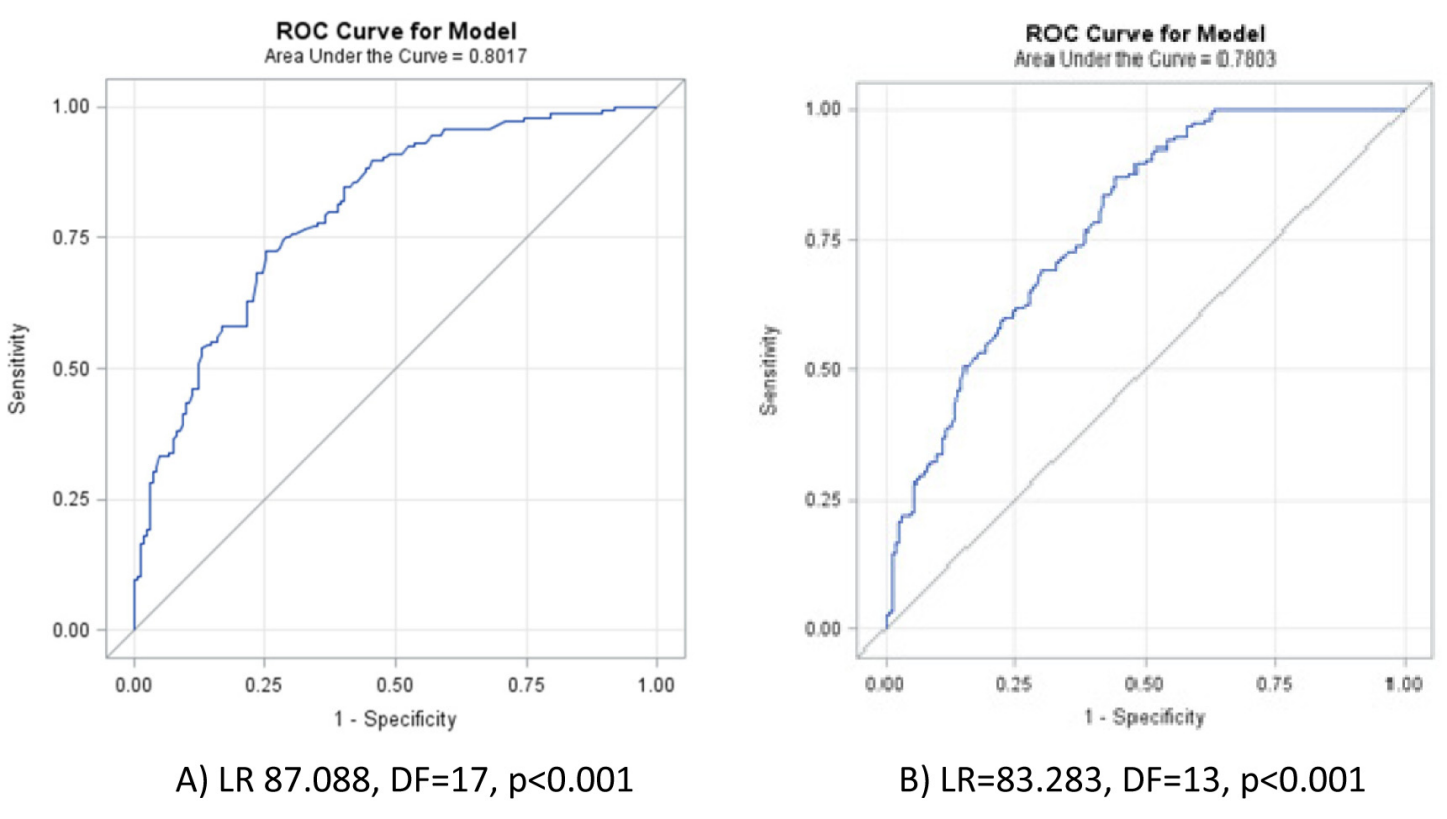
Receiver-operating characteristic curves for Systemic Lupus International Collaborating Clinics (SLICC)-12 (likelihood ratio = 87.088, degrees of freedom = 17, *P* < 0.001) (**A**) and American College of Rheumatology (ACR)-97 criteria (likelihood ratio = 83.283, degrees of freedom = 13, *P* < 0.001) (**B**). ROC – receiver-operating characteristic.

**Figure 3:**
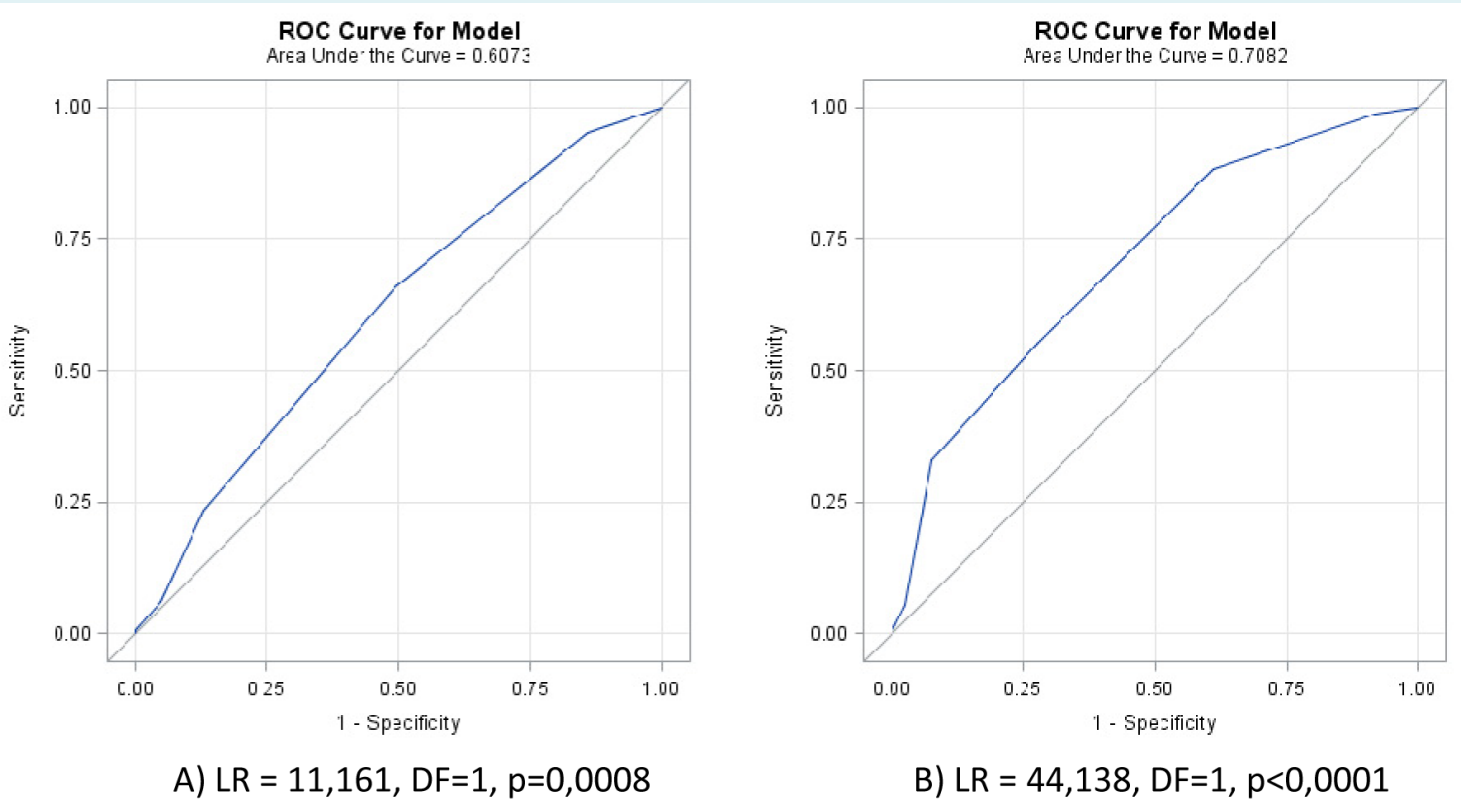
Receiver-operating characteristic curves for Systemic Lupus International Collaborating Clinics (SLICC)-12 criteria – clinical (likelihood ratio = 11.161, degrees of freedom = 1, *P* = 0.0008) (**A**) vs immunologic (likelihood ratio = 44.138, degrees of freedom = 1, *P* < 0.0001) (**B**). ROC – receiver-operating characteristic.

Univariate analysis showed that the SLICC-12 classification was a better predictor of SLE than the ACR-97 criteria, with the areas under the ROC-curve (AUC)_SLICC_ = 0.801 and AUC_ACR_ = 0.780 (Figures 2A and 2B). We found a moderate relationship of the number of ACR-97 criteria with the SLE diagnosis, with AUC = 0.711. An increase in the total number of ACR-97 criteria by 1 increased the odds ratio (OR) for diagnosing SLE by almost 3 times (OR 2.916, 95% confidence interval [CI] 1.085-4.078). The total number of SLICC-12 criteria was a slightly better predictor for diagnosing SLE than the number of ACR-97 criteria, with AUC = 0.728. Interestingly, an increase in the number of SLICC-12 criteria by 1 increased the OR for diagnosing SLE by almost 2 times (OR 1.727, 95% CI 1.454-2.050). When we analyzed the clinical and immunologic SLICC criteria separately, immunologic criteria showed greater predictive value, with AUC_immun_ = 0.708 and AUC_clin_ = 0.607 (Figures 3A and 3B) (Table 4 and Table 5).

**Table 4:** Logistic regression – the analysis of effects for ACR-97 criteria*

ACR-97 Criteria	DF	Wald χ^2^	*P*	OR	95% CI	c (multivariate analysis) = 0.7803	c (univariate analysis)
**Butterfly rash**	1	6.1690	0.0130	2.619	1.225-5.597	0.518
**Discoid rash**	1	10.5618	0.0012	4.469	1.840-11.743	0.503
**Oral ulcers**	1	1.1994	0.2734	2.263	0.467-14.739	0.501
**Photosensitivity**	1	5.2923	0.0214	2.354	1.135-4.880	0.512
**Arthritis**	1	1.6436	0.1998	1.523	0.801-2.897	0.507
**Serositis**	1	3.7244	0.0536	5.651	0.973-32.812	0.521
**Renal disorder**	1	7.0237	0.0080	4.170	1.450-11.988	0.543
**NPSLE**	1	2.6114	0.1061	4.009	0.744-21.601	0.511
**Hematologic disorder**	1	4.9577	0.0260	1.946	1.083-3.498	0.505
**Immunologic disorder**	1	43.4587	<0.0001	10.933	5.369-22.263	0.688
**ANA**	1	2.5167	0.1126	2.222	0.829-5.957	0.550
**LR = 83.283, DF = 13, *P* < 0.0001**							

*ACR-97 – American College of Rheumatology classification from 1997; NPSLE – neuropsychiatric lupus erythematosus; ANA – anti-nuclear antibody; LR – likelihood ratio; DF – degrees of freedom; OR – odds ratio; 95% CI – 95% confidence interval; c – area under the ROC curve.

**Table 5:** Logistic regression – the analysis of effects for SLICC-12 criteria*

Criteria	DF	Wald χ^2^	*P*	OR	95% CI	c (multivariate analysis) = 0.8017	c (univariate analysis)
**ACL**	1	2.6327	0.1047	1.615	0.905-2.883	0.502
**CCL**	1	11.9335	0.0006	4.845	1.979-11.862	0.506
**Oral ulcers**	1	0.9115	0.3397	2.090	0.460-9.487	0.505
**Alopecia**	1	0.1323	0.7161	0.830	0.304-2.264	0.507
**Sinovitis**	1	5.7563	0.0164	2.019	1.137-3.584	0.558
**Serositis**	1	2.2970	0.1296	3.576	0.688-18.576	0.528
**Renal disorder**	1	6.9346	0.0085	4.196	1.443-12.203	0.540
**NPSLE**	1	0.2949	0.5871	1.225	0.590-2.544	0.525
**Hemolytic anemia**	1	0.5408	0.4621	4.286	0.089-207.265	0.507
**L/ly-penia**	1	2.0488	0.1523	1.514	0.858-2.672	0.507
**Thrombocytopenia**	1	0.0582	0.8093	0.902	0.389-2.088	0.503
**ANA**	1	1.9760	0.1598	1.992	0.762-5.206	0.540
**Anti-dsDNA**	1	30.8423	<0.0001	5.131	2.881-9.139	0.695
**Anti-Sm**	1	0.1703	0.6798	0.830	0.342-2.011	0.536
**APA**	1	9.7391	0.0018	2.596	1.426-4.725	0.607
**Complement**	1	0.6989	0.4032	1.259	0.733-2.163	0.561
**Coombs test**	1	2.7419	0.0977	4.259	0.766-23.671	0.521
**LR = 87.088, DF = 17, *P* < 0.0001**							

*SLICC-12 – Systemic Lupus International Collaboration Clinics classification from 2012; ACL –acute cutaneous lupus; CCL – chronic cutaneous lupus; NPSLE – neuropsychiatric lupus erythematosus; L – leukocyte; ly – lymphocyte; ANA – anti-nuclear antibody; dsDNA – double stranded DNA; anti-Sm – anti-Smith antibody; APA – anti-phospholipid antibody; LR – likelihood ratio; OR – odds ratio; 95% CI – 95% confidence interval; c – area under the ROC curve.

**Figure 4:**
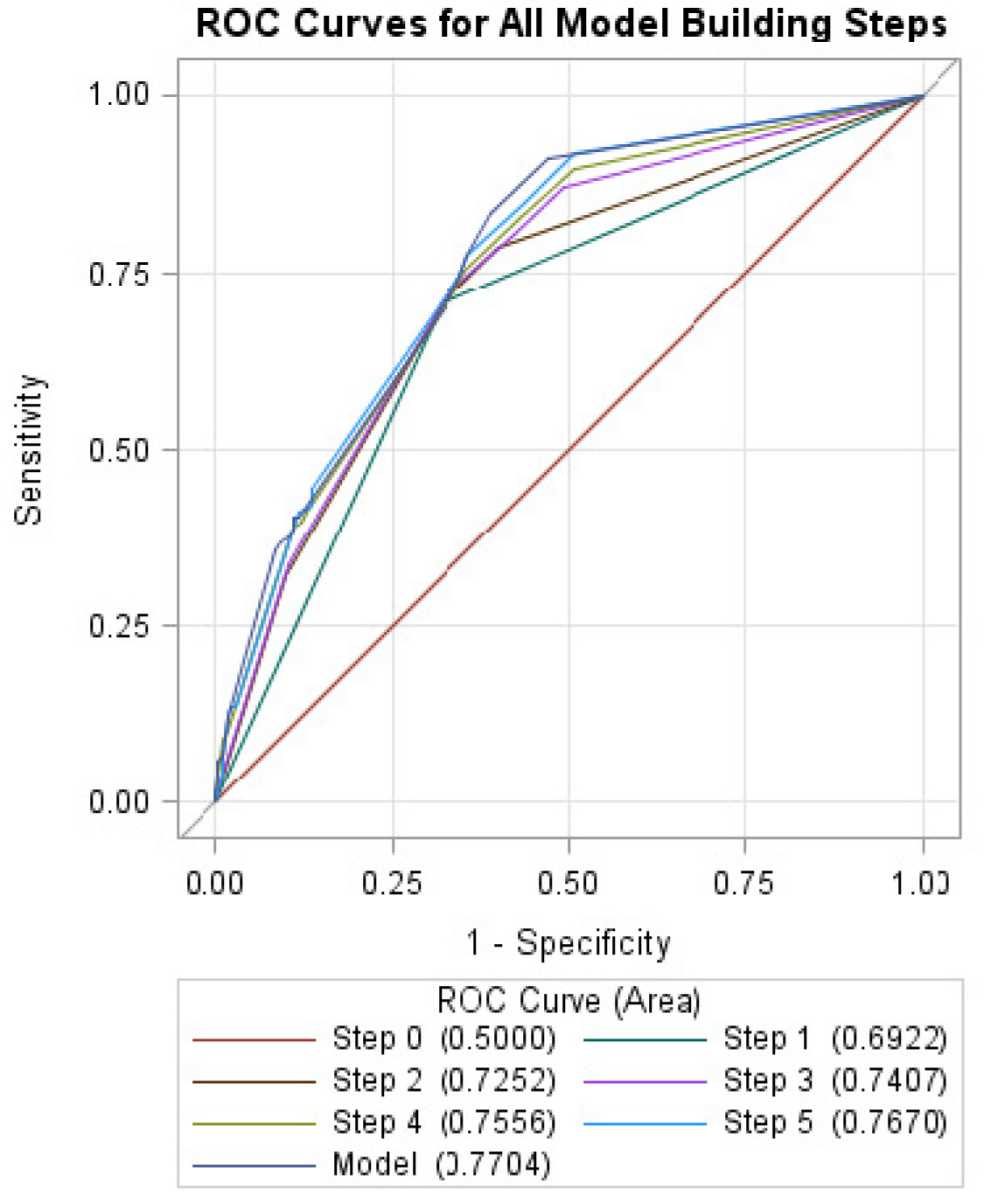
The best predictive model for systemic lupus erythematosus, generated by stepwise procedure from the Systemic Lupus International Collaborating Clinics (SLICC)-12 criteria, area under the receiver-operating characteristic (ROC) curve = 0.770. Step 0 – anti-dsDNA antibody, Step 1 – anti-phospholipid antibodies, Step 2 – chronic cutaneous lupus, Step 3 – renal impairment, Step 4 – coombs test, Step 5 – anti-β2-glycoprotein I.

**Figure 5:**
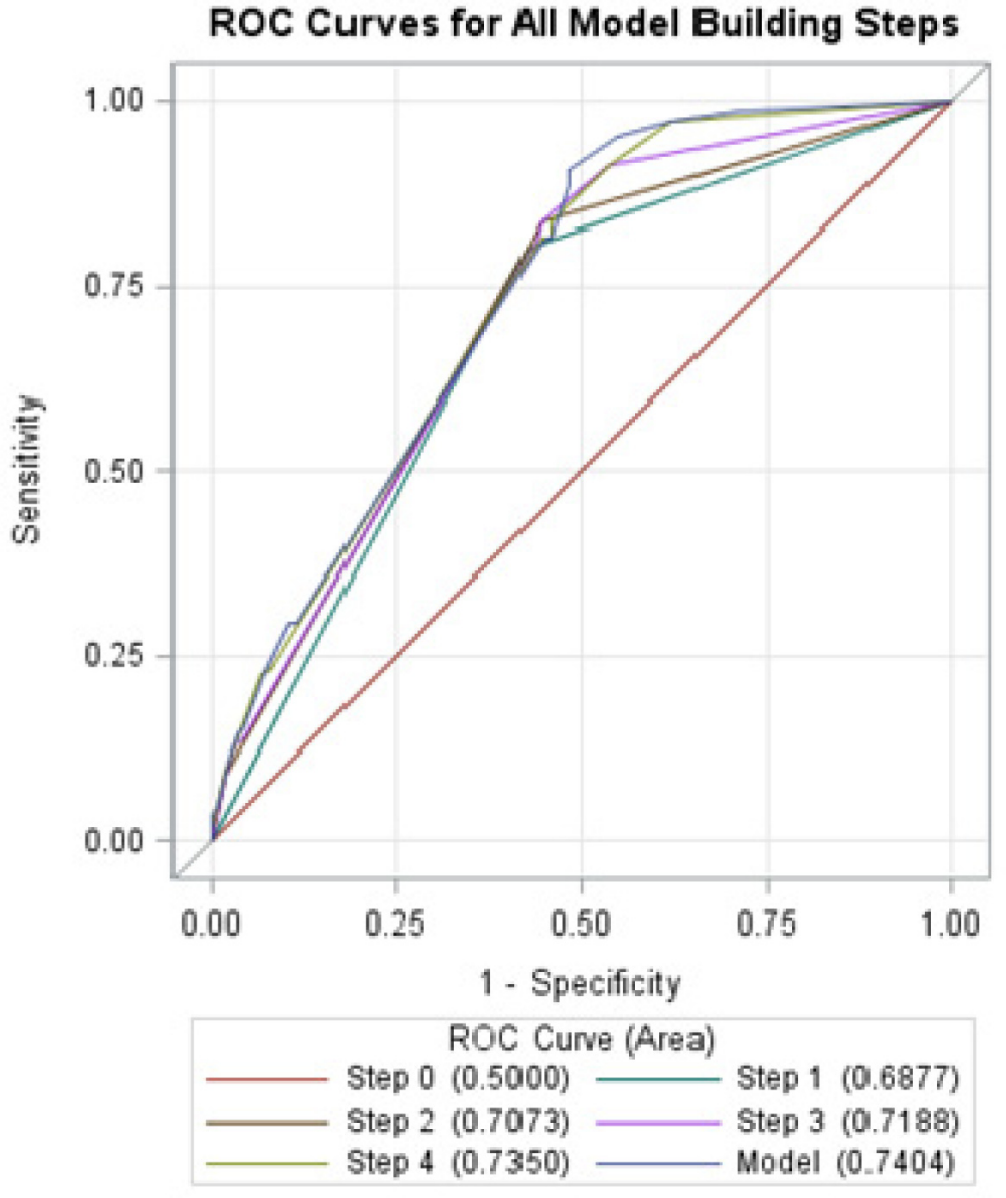
The best predictive model for systemic lupus erythematosus, generated by stepwise procedure from the American College of Rheumatology (ACR)-97 criteria, area under the receiver-operating characteristic (ROC)curve = 0.735. Step 0 – immunologic disorder, Step 1 – renal impairment, Step 2 – discoid rash, Step 3 – butterfly rash, Step 4 – photosensitivity.

Using stepwise procedure, we obtained the best predictive models for SLE. Stepwise procedure for SLICC-12 classification generated a combination of 6 criteria: chronic cutaneous lupus, renal impairment, anti-dsDNA antibody, anti-cardiolipin antibody, β2-glycoprotein I, and a positive Coombs test. Taken together, these criteria had the best predictive value for SLE, with AUC = 0.770 (Figure 4). Stepwise procedure for the ACR-97 classification generated a model containing butterfly rash, discoid rash, photosensitivity, renal impairment, and immunologic disorder. The latter model was the best predictor of SLE in our patient cohort, with AUC = 0.740 (Figure 5).

## Discussion

Our results show the superiority of the SLICC-12 criteria and their somewhat greater predictive value for diagnosing SLE compared with the ACR-97 criteria, especially in the early disease stage. Better defined clinical features within each criterion contribute to higher sensitivity of the SLICC-12 classification. Nevertheless, in our cohort, the specificity of SLICC-12 classification was too low and decreased with SLE duration. The ACR-97 classification showed higher specificity than the SLICC-12 classification. Our results agree with the results of criteria validation by the SLICC group and other research, which have shown high specificity of the ACR-97 criteria in the established disease, with lower, but acceptable sensitivity (9,26,28,29). SLE diagnosis is hard to confirm due to its heterogeneous clinical presentation and slow accumulation of symptoms. Therefore, the ACR-97 criteria do not have adequate sensitivity to recognize patients in the early disease stage. On the other hand, SLICC-12 criteria have so far shown higher sensitivity than the ACR-97 in the early disease, as well as in milder SLE cases, in UCTD, and in juvenile SLE (24-26,29-34). Nevertheless, in our study, SLICC-12 classification often had lower specificity than the ACR-97, which was also confirmed in the validation process by the SLICC group (14).

When we compared the two classifications in terms of disease duration, the greatest difference in sensitivity emerged in the early disease stage. These results agree with those from other reports. A study involving 2055 patients from Spanish and Portuguese registers (26) showed that the SLICC-12 criteria had significantly higher sensitivity than the ACR-97 criteria in the earlier disease stages and that their sensitivity increased with disease duration. In the long-standing disease, the sensitivity of the two classifications did not significantly differ (26). Studies on pediatric patients with SLE also showed higher sensitivity in the earlier disease stages and lower specificity (31-33). A study involving patients from a Swedish lupus register reported that SLICC-12 criteria had higher sensitivity (94% vs 90%), but a surprisingly low specificity (74%). The authors recommend that a combination of the two criteria sets is used to define patients more precisely (28). Furthermore, research on 495 patients from 12 Japanese medical centers showed similar results: higher sensitivity (99% vs 88%) and lower specificity of the SLICC-12 criteria compared with the ACR-97 criteria (80% vs 85%) (35). In the Mexican population, fewer patients were missclassified when the ACR-97 criteria were used, with higher specificity and positive predictive value (36). This implies that the ACR-97 criteria are more reliable in a “real-life” setting. A study conducted in Olmsted county (Minnesota, USA) showed a higher SLE incidence if the SLICC-12 criteria are used, mostly because of the added classification rule on biopsy-proven lupus nephritis along with positive ANA or anti-dsDNA antibodies (37).

Regression analysis in our patient cohort showed the superioritiy of the SLICC-12 classification. The number of the SLICC-12 criteria met by a patient was more strongly associated with SLE diagnosis than the number of ACR-97 criteria. Nevertheless, the increase in the number of the ACR-97 criteria met was more strongly associated with SLE. Univariate analysis showed a minor difference between the two classifications. In general, both criteria sets in our patient cohort showed significant association with SLE, with SLICC-12 classification being a slightly better predictor. Multivariate analysis showed different predictive value of some criteria in the two classifications. The SLICC criteria that most contributed to diagnosing SLE were chronic cutaneous lupus, synovitis, renal impairment, anti-dsDNA, and APL antibodies. On the other hand, ACR-97 criteria that most contributed to diagnosing SLE were acute cutaneous lupus (photosensitivity and butterfly rash), discoid lupus, serositis, renal impairment, hematologic, and immunologic disorder. Acute cutaneous lupus in the new criteria encompasses various manifestations that often overlap and, taken together, in our cohort did not show enough specificity for SLE. Therefore, they are not as represented as the often described malar rash and photosensitivity in the ACR-97 criteria. As opposed to earlier research (37), the two classifications did not significantly differ regarding renal impairment in our cohort. Given that arthritis/synovitis is less strictly defined in SLICC-12 criteria, it emerged significant in regression analysis, as well as the hematologic criterion of the ACR-97 classification, which incorporates a disorder in all three blood cell lineages. Although stepwise procedure generated optimal criteria combinations for predicting SLE, the strongest predictor turned out to be the overall classification, regarding both SLICC-12 and ACR-97 classification. This is contradictory to the results of Mesa at al (38), who, also employing regression analysis, reported that reduced models, rather than the whole classification, were better discriminators of SLE patients among unclear cases of MCTD. Al-Daabil et al (39) found that strong predictors of SLE were oral ulcerations, renal impairment, and anti-dsDNA antibodies. A study involving 110 patients with SLE from another tertiary center in Croatia reported a linear correlation of the number of SLICC-12 criteria and disease activity, while no such correlation was reported for the ACR-97 criteria (40). Amezcua-Guerra et al (36) showed high positive and negative predictive values for both classifications, with higher specificity of the ACR-97 criteria for SLE.

Our study has several limitations. First, it was carried out in a single center and, therefore, may not account for the entire population with SLE in Croatia. Nevertheless, our center has a substantial number of SLE patients, from all parts of the country (predominantly northwestern). Second, a considerable number of patients (35%) in this study was in the early stage of the disease at the time of data collection. This could imply a milder disease in the overall sample. Still, taken together, all studies carried out so far, as well as this study, have shown overall a minor difference between the two classifications. SLICC-12 classification, despite its high sensitivity, wider range of incorporated clinical manifestations, and immunologic criteria, does not show significant supremacy in a real-life setting. This is largely due to the high specificity of the ACR-97 criteria. Due to relatively low sensitivity, classification criteria are not an appropriate diagnostic tool in routine clinical setting. Their primary purpose is defining homogeneous patient cohorts for clinical research. Therefore, the gold standard for diagnosing most of rheumatic diseases, including SLE, remains an experienced rheumatologist's assessment. The results of this research contribute to the ongoing imitative for developing improved classification criteria for SLE (41).
